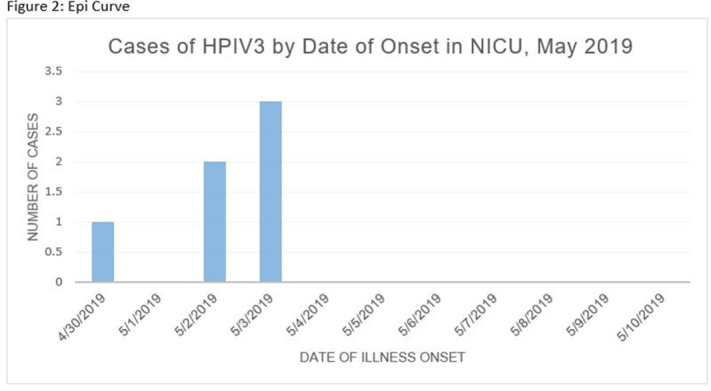# Successful Control of Human Parainfluenza Type 3 Outbreak in a Level IV Neonatal Intensive Care Unit

**DOI:** 10.1017/ash.2021.48

**Published:** 2021-07-29

**Authors:** Bhagyashri Navalkele, Sheila Fletcher, Sanjosa Martin, Regina Galloway, April Palmer

## Abstract

Human parainfluenza (HPIV) is a common cause for upper respiratory tract illnesses (URTI) and lower respiratory tract illnesses (LRTI) in infants and young children. Here, we describe successful control of an HPIV type 3 (HPIV3) outbreak in a neonatal intensive care unit (NICU). NICU babies with new-onset clinical signs or symptoms of RTI and positive HPIV-3 nasopharyngeal specimen by respiratory pathogen panel (RPP) test on hospital day 14 or later were diagnosed with hospital-onset (HO) HPIV-3 infection. After 3 NICU babies were diagnosed with HO HPIV-3, an outbreak investigation was initiated on May 3, 2019, and continued for 2 incubation periods since the last identified case. Enhanced infection prevention measures were immediately implemented. All positive cases were placed in a cohort in a single pod of the NICU and were placed on contact precautions with droplet isolation precautions. Dedicated staffing and equipment were assigned. Environmental cleaning and disinfection with hospital-approved disinfectant wipes was performed daily. Visitors were restricted in the NICU. All employees entering the NICU underwent daily symptom screening for respiratory tract illness. All NICU babies were screened daily for respiratory tract illness with prompt isolation and RPP testing on positive screen. To determine the source of the HPIV3 outbreak, all HPIV3-positive specimens from the NICU and available temporally associated community-onset (CO) controls collected from non-NICU units were sent to the Centers for Disease Control and Prevention (CDC) for whole-genome sequencing (WGS) analysis. The first and last cases of HPIV-3 were diagnosed on May 1 and May 5, 2019, respectively. In total, 7 HO HPIV3 cases were reported: 1 in newborn nursery (NBN) and 6 in NICU. The case from the NBN was determined to be unrelated to the outbreak and the source was linked to a sick visitor. Of the 6 NICU babies, 5 had an LRTI and 1 had a URTI. Average time from admission to diagnosis was 71 days (range, 24–112). None had severe illnesses requiring intubation, and all had full recovery. No CO HPIV3 cases were reported from the NICU during the investigation. A maximum likelihood phylogenetic tree of HPIV3 WGS (Figure 1) showed that sequences from the 6 HO cases clustered together separately from the 3 CO controls, suggesting a single source of transmission, and 3 CO cases were not related to the HO cases or source of the outbreak. Early diagnosis and isolation of respiratory tract viral infections is important to prevent an outbreak. Successful control of outbreak in NICU requires prompt implementation of infection prevention measures with focus on symptom screening, cohorting, and disinfection practices.

**Funding:** No

**Disclosures:** None

**Figure 1.**
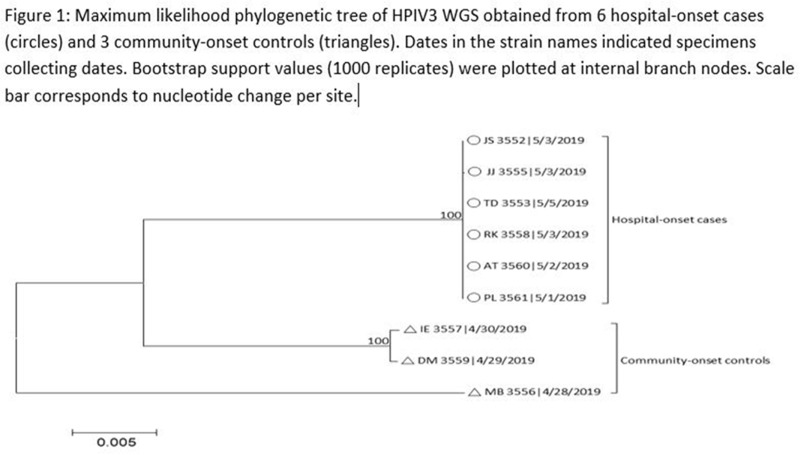

**Figure 2.**